# Long-term prognosis of chronic depression in adolescence

**DOI:** 10.1192/j.eurpsy.2024.740

**Published:** 2024-08-27

**Authors:** V. Kaleda, V. Migalina

**Affiliations:** ^1^Mental Health Research Centre, Moscow, Russian Federation

## Abstract

**Introduction:**

juvenile chronic depression is characterized by high prevalence, difficulties in diagnosis, nosological qualification and prognostic assessment. According to epidemiological data, the frequency of these conditions ranges from 1.5% to 3% in the general population (Gutiérrez-Rojas et al. Braz. J. Psychiatr 2020; 42 657-672), and among all depressions in adolescence, a chronic course develops in about 20% of cases (Blanco C., 2010 et al. The J clinical psychiatry 2010; 71(12) 6501). Due to the polymorphism of the clinical picture and the peculiarities of juvenile ontogenesis, difficulties arise in nosological and prognostic assessment.

**Objectives:**

to study the long-term prognosis of chronic depression, depending on the variant of its course.

**Methods:**

Catamnestic examination was performed on 64 patients of adolescent age (16-25 years), for chronic depressive state lasting more than two years (F31.3, F31.4, F32 (except F32.3), F33 (except F33.3), F34, F34.1, F21, F20 according to ICD-10). The duration of the catamnesis is more than 10 years. The PSP scale was used for psychometric assessment.

**Results:**

when analyzing the ten-year course of juvenile chronic depression, three variants were identified: regredient (23.4%), monotonous (35.9%) and progredient (40.6%). The regredient course was characterized by a marked reduction or disappearance of psychopathological disorders with the formation of further remission with a high level of functioning in all spheres of life and complete social and labor adaptation (81-100 points on the PSP scale). The monotonous course was characterized by low variability and insignificant dynamics of individual manifestations throughout the disease with the preservation or some decrease in the level of educational and labor adaptation with the restoration of previous social contacts and a fairly high quality of life (scores 61-80 on the PSP scale). The progressive course was characterized by the gradual addition of new psychopathological disorders, or the aggravation of existing ones, patients had a distinct decrease in educational, labor and social adaptation (scores 50-31 on the PSP scale) or complete maladaptation of all spheres of life (scores <40 on the PSP scale).

**Conclusions:**

The high incidence of progressive and monotonous course in juvenile chronic depression, contributing to a decrease in the level of functioning of patients, indicates the importance of timely detection of these conditions and the need for careful selection of therapy.

**Disclosure of Interest:**

None Declared

